# Glioma cell fate decisions mediated by Dll1-Jag1-Fringe in Notch1 signaling pathway

**DOI:** 10.1186/s12918-017-0457-6

**Published:** 2017-09-21

**Authors:** Xiaofei Shi, Ruiqi Wang

**Affiliations:** Department of Mathematics, Shanghai University, No.99, Shangda Road, Shanghai, 200444 China

**Keywords:** Notch, Fringe, Gliomas, Trans-activation, Cis-inhibition

## Abstract

**Background:**

The Notch family of proteins plays a vital role in determining cell fates, such as proliferation, differentiation, and apoptosis. It has been shown that Notch1 and its ligands, Dll1 and Jag1, are overexpressed in many glioma cell lines and primary human gliomas. The roles of Notch1 in some cancers have been firmly established, and recent data implicate that it plays important roles in glioma cell fate decisions. This paper focuses on devising a specific theoretical framework that incorporates Dll1, Jag1, and Fringe in Notch1 signaling pathway to explore their functional roles of these proteins in glioma cells in the tumorigenesis and progression of human gliomas, and to study how glioma cell fate decisions are modulated by both trans-activation and cis-inhibition.

**Results:**

This paper presents a computational model for Notch1 signaling pathway in glioma cells. Based on the bifurcation analysis of the model, we show that how the glioma cell fate decisions are modulated by both trans-activation and cis-inhibition mediated by the Fringe protein, providing insight into the design and control principles of the Notch signaling system and the gliomas.

**Conclusions:**

This paper presents a computational model for Notch1 signaling pathway in glioma cells based on intertwined dynamics with cis-inhibition and trans-activation involving the proteins Notch1, Dll1, Jag1, and Fringe. The results show that how the glioma cell fate transitions are performed by the Notch1 signaling. Transition from grade III ∼ IV with significantly high Notch1 to grade I ∼ II with high Notch1, and then to normal cells by repressing the Fringe levels or decreasing the strength of enhancement induced by Fringe.

## Background

Notch signaling pathway is an evolutionarily conserved cell-cell communication mechanism governing cell fate decisions during cell development. The signaling pathway includes the Notch transmembrane receptor and its ligands Delta and/or Jagged [1–3]. The Notch inactivation within the same cell is termed as cis-inhibition, which leads to the degradation of both proteins, therefore not generating a signal. The Notch receptor of one cell binds with a Notch ligand of its neighboring cells, i.e., trans-activation, leads to the formation of an active intracellular domain called Notch intracellular domain (NICD), which can translocate to the nucleus and initiate transcription of its target genes [4]. It has been shown that the trans-activation and cis-inhibition play important roles in cell fate decisions, such as neural fate decisions [5].

With only a single type of ligand and a single type of receptor it is relatively straightforward to evaluate Notch signaling’s effect. To date, four Notch receptors have been identified (Notch 1-4) in humans, with five canonical ligands including three members of the Delta family (Dll1, Dll3, Dll4) and two members of the Serrate family (Jag1 and Jag2, homologues of Drosophila Serrate) [6]. At the same time, the family of Fringe-related proteins is a major Notch regulator, which can promote or suppresse Notch signaling, depending on the Notch ligands [7, 8]. There is only a single Fringe in Drosophila, while there are three homologues in mammals: Lunatic Fringe (LFng), Manic Fringe (MFng) and Radical Fringe (RFng) [6]. Of the three mammalian Fringe proteins, it has been shown that only LFng can enhance Notch1 signaling induced by Dll1 and suppress the signaling induced by Jag1 in coculture reporter assays [9]. It has been also shown that MFng can suppress Jag1 induced signaling through Notch1, while the effects of MFng on Notch1 signaling in response to Dll1 have not been reported [10]. Given the evolutionary conservation of the Notch pathway, three Fringe proteins in human have also been identified [11].

The Notch family of receptors consists of heterodimeric transmembrane proteins intimately involved in the determination of cell fate. Notch signaling can play a positively or negatively role in processes of proliferation, differentiation, and apoptosis, depending on the cell type [12, 13]. Alagille’s syndrome in humans, marked by cholestasis/jaundice, characteristic facies, and arterial defects, has been traced to a defect in Jag1 [14, 15]. Dll1 and Jag1 have been found to be up-regulated in cervical cancers [16]. More recently, it has been shown that the Jag1 intracellular domain can up-regulate the activator protein 1 (AP-1) activity [17], a signaling pathway known to be important in many cancers.

To date, it has been shown that Notch1 and its ligands, Dll1 and Jag1, are overexpressed in many glioma cell lines and primary human gliomas. Immuno-histochemistry of a primary human glioma tissue array shows the presence in the nucleus of the Notch1 intracellular domain, indicating Notch1 activation in situ. Down-regulation of Notch1, Dll1, or Jag1 by RNA interference induces apoptosis and inhibits proliferation in multiple glioma cell lines [18]. Glioma is the most common clinical central nervous system malignancies. The patients with glioma have poor effects. The average survival time is short [19]. It has been demonstrated that Notch1 mRNA in human brain gliomas and normal brain tissue can be expressed, but the expression in human gliomas was significantly higher than in normal brain tissue, indicating that the expression levels of Notch1 may be associated with human glioma tumorigenesis and development. Gliomas are divided into four levels: grade I ∼ II and grade III ∼ IV. The expression of Notch1 mRNA in human gliomas is significantly higher than in normal brain tissue, and the level of Notch1 mRNA in III ∼ IV is significantly higher than that of grade I ∼ II, wihch indicates the expression levels of Notch1 is associated with not only pathological grade of gliomas, but also the degree of malignancy of gliomas [20]. As for standard therapies, such as chemotherapy, surgery, and radiation, have had limited success in treating patients with high-grade gliomas. Existing results show that the cancer cells may depend on a single Notch ligand and they further suggest a potential Notch juxtacrine/autocrine loop in gliomas [18]. Therefore, Notch1 and its ligands may present novel therapeutic targets in the treatment of gliomas.

The purpose of this paper is to present a computational model for Notch1 signaling pathway in glioma cell lines and primary human gliomas based on intertwined dynamics with cis-inhibition and trans-activation involving the proteins Notch1, Dll1, Jag1, and Lunatic Fringe. Mathematical models of Notch signaling, with different levels of sophistication, have been proposed for different organisms for which sufficient knowledge of molecular biology exists. All these models can produce different results but are not sufficient in several important respects. First, most of the previous models do not include an essential characteristic of Notch signaling, i.e. cis-inhibition [21, 22]. Second, even when cis-inhibition is incorporated, its link to glioma cell lines and primary human gliomas and its effects on cell fate decisions have not been well considered [23]. Most models focus on how Notch signaling plays different roles in various cell fate decisions, but how Fringe affects the fate decisions in glioma cell lines has not been well investigated. Thus, a new model needs to be developed so as to investigate the combinatorial effects of cis-inhibition, trans-activation, and Fringe regulation on glioma cell fate decisions, their operating mechanisms, and potential implications in the treatment of gliomas.

## Methods

**Fig. 1:**
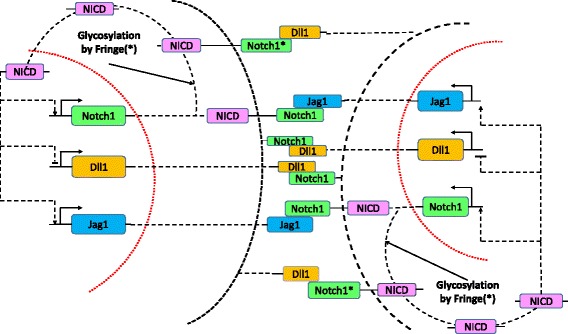
System for signal integration in the intracellular and intercellular Notch1 signaling pathway. Notch1, the transmembrane receptor of one cell, binds to Dll1 or Jag1, the transmembrane ligands belonging to the neighboring cell. This trans-interaction leads to the cleavage and release of NICD that regulates the production of the two ligands asymmetrically, i.e., it inhibits Dll1 but activates Jag1. Interaction between Notch1 receptor and ligands (Dll1 or Jag1) of the same cell (cis-interaction) leads to the degradation of both the receptor and the ligands. Glycosylation of Notch1 by Fringe modifies Notch1 to have a higher affinity for binding to Dll1 and a lower affinity for binding to Jag1

**Fig. 2:**
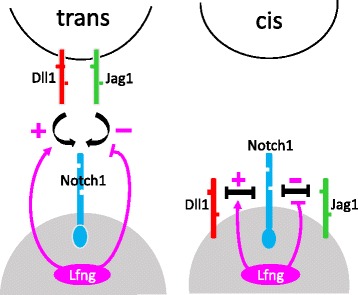
Regulation diagram. Notch1 could interact by cis and trans with Jag1 and Dll1 ligands, and in which one of the Fringe-related proteins, Lunatic Fringe (LFng), could modulate these interactions. LFng modification of Notch1 enhances trans activation from Dll1 and weakens trans-activation from Jag1 (*left*). LFng modification of Notch1 enhances cis-interactions with Dll1 and weakens cis-interactions with Jag1 (*right*). Interactions are indicated by + and − for positive and negative regulation, respectively

The regulatory processes between Notch1, Jag1, Dll1, and Fringe are schematized in Fig. 1. Notch1 signaling pathway is involved in glioma stem cells proliferation and differentiation. It has been shown that Notch1 protein is over expressed in human gliomas [19]. Notch1 signaling pathway, including the processes of cis-inhibition, trans-activation, and the regulation mediated by Lunatic Fringe is shown in Fig. 2. It is known that Fringe may play an important role in the treatment of gliomas.

For gliomas, most researchers are currently engaged in the study on related factors of Notch signaling pathway [7, 19], they pay less attention to the relationship between the related factors in terms of mathematical theory. The model presented here involves several aspects. First, the Notch1 binds to Dll1 or Jag1 with the same affinity when the regulation mediated by Fringe is not incorporated. Second, when the Fringe regulation on pathway is incorporated, it can increase the Notch1-Dll1 binding affinity and decrease the Notch1-Jag1 binding affinity. We mainly consider the two-cell system, and the system can be extended to the case where each cell has *j*-neighbors.

The basic model of Notch signaling incorporating the cis-inhibition and trans-activation was previously developed [24]. Subsequent model by incorporating Jag1 in addition to Delta and the asymmetric effect of NICD which activates Notch and Jag1 but represses Delta was also proposed [25]. Trans-interaction leads to the release of the NICD signal into the cytoplasm, resulting in subsequent activation of downstream target genes, while cis-interaction leads to the degradation of both proteins, Notch and Delta, therefore no generation of any signal. Under the assumption that the affinity of Notch1 to Dll1 or Jag1 is the same, when the Fringe regulation is ignored, the dynamics for the Notch1 receptor (N), the ligands Dll1 (D) and Jag1 (J), and the signal NICD (I) are given by the following equations

1 $$\begin{array}{*{20}l} \frac{dN}{dt}&=\left(1+\frac{I^{2}}{I^{2}+I_{0}^{2}}\right)N_{0}-k_{c}N(D+J)\\ &\quad-k_{t}N(D_{ext}+J_{ext})-\gamma N, \end{array} $$

2 $$\begin{array}{*{20}l} \frac{dD}{dt}&=\frac{I_{0}^{2}}{I^{2}+I_{0}^{2}}D_{0}-k_{c}DN-k_{t}{DN}_{ext}-\gamma D, \end{array} $$

3 $$\begin{array}{*{20}l} \frac{dJ}{dt}&=\left(1+\frac{I^{5}}{I^{5}+I_{0}^{5}}\right)J_{0}-k_{c}JN-k_{t}{JN}_{ext}-\gamma J, \end{array} $$

4 $$\begin{array}{*{20}l} \frac{dI}{dt}&=k_{t}N(D_{ext}+J_{ext})-\gamma_{I}I, \end{array} $$

where *N*_0_,*D*_0_, and *J*_0_ are the innate production rates of Notch1, Dll1, and Jag1, respectively. *γ* represents the degradation rate of all three transmembrane proteins Notch1, Jag1, and Dll1, which are assumed to be the same. *N*_*ext*_,*D*_*ext*_, and *J*_*ext*_ represent the amount of protein available for binding from neighboring cells. *k*_*c*_ and *k*_*t*_ represent the strengths of cis-inhibition and trans-activation, respectively. *γ*_*I*_ stands for the degradation rate of NICD.

Glycosylation of Notch1 by Fringe modulates the binding affinity of the two ligands to Notch1. The glycosylated Notch1 has a higher binding affinity for Dll1 but lower affinity to bind to Jag1, compared to the unglycosylated Notch1 [6]. Thus, to incorporate this mechanism to our model, while representing effective Notch1 in gliomas cell (sum of glycosylated and unglycosylated Notch1), the model can be rewritten as

5 $$\begin{array}{*{20}l} \frac{dN}{dt}&=\left(1+\frac{I^{2}}{I^{2}+I_{0}^{2}}\right)N_{0}-\left(1+\frac{a [L]^{n}}{k_{1}^{n}+[L]^{n}}\right)\left(k_{c}ND\right.\\ &\quad\left.+k_{t}{ND}_{ext}\right)-\frac{k_{2}^{n}}{k_{2}^{n}+[L]^{n}}(k_{c}NJ+k_{t}{NJ}_{ext})-\gamma N, \end{array} $$

6 $$\begin{array}{*{20}l} \frac{dD}{dt}&=\frac{I_{0}^{2}}{I^{2}+I_{0}^{2}}D_{0}-\left(1+\frac{a [L]^{n}}{k_{1}^{n}+[L]^{n}}\right)\\ &\quad\times(k_{c}DN+k_{t}{DN}_{ext})-\gamma D, \end{array} $$

7 $$\begin{array}{*{20}l} \frac{dJ}{dt}&=\left(1+\frac{I^{5}}{I^{5}+I_{0}^{5}}\right)J_{0}-\frac{k_{2}^{n}}{k_{2}^{n}+[L]^{n}}\\ &\quad\times(k_{c}JN+k_{t}{JN}_{ext})-\gamma J, \end{array} $$

8 $$\begin{array}{*{20}l} \frac{dI}{dt}&=\left(1+\frac{a [L]^{n}}{k_{1}^{n}+[L]^{n}}\right) k_{t}{ND}_{ext}\\ &\quad+\frac{k_{2}^{n}}{k_{2}^{n}+[L]^{n}}k_{t}{NJ}_{ext}-\gamma_{I}I, \end{array} $$

where *L* stands for the Fringe. Hill functions are used to show the effects of Fringe on cis-inhibition and trans-activation. The definitions of these parameters, *N*_0_,*D*_0_,*J*_0_, *k*_*c*_, *k*_*t*_, *γ*, and *γ*_*I*_, are the same as the model (1)-(4). The standard values of all parameters are listed in Table 1. In the two cell model, the adjacent cell means the other cell. But for the hexagonal cell arrangement, the adjacent cells mean the six immediate neighbors, the sum of the expression levels in adjacent cells is divided by six. In order to extend to the multiple cell model, we consider the case where cell *i* (*i*=1,…,*n*) has *j*-neighbors. These regulatory processes can be expressed by a set of ordinary differential equations as follows

9 $$\begin{array}{*{20}l}{} \frac{{dN}_{i}}{dt}&=\left(1+\frac{I_{i}^{2}}{I_{i}^{2}+I_{0}^{2}}\right)N_{0}- \left(1+\frac{a [L]^{n}}{k_{1}^{n}+[L]^{n}}\right)(k_{c}N_{i}D_{i}\\ &\quad +k_{t}N_{i}\left\langle D_{j}\right\rangle_{i})-\frac{k_{2}^{n}}{k_{2}^{n}+[L]^{n}}(k_{c}N_{i}J_{i}+k_{t}N_{i} \left\langle J_{j}\right\rangle_{i})-\gamma N_{i}, \end{array} $$

**Table 1:** Standard parameter values in the model (5)−(8)

Parameters	Definitions	Values	Unit
*N*_0_	The innate production rates of Notch1	1400	Number of proteins
*D*_0_	The innate production rates of Dll1	1600	Number of proteins
*J*_0_	The innate production rates of Jag1	1200	Number of proteins
*I*_0_	The innate production rates of NICD	200	Number of proteins
*γ*	The degradation rate of proteins	0.1	time ^−1^(*h* ^−1^)
	Notch1, Jag1, and Dll1		
*γ*_*I*_	The degradation rate of NICD	0.5	time ^−1^(*h* ^−1^)
*k*_*t*_	The strengths of trans-activation	4.7×10^−5^	time ^−1^(*h* ^−1^)
*k*_*c*_	The strengths of cis-activation	6.1×10^−4^	time ^−1^(*h* ^−1^)

**Fig. 3:**
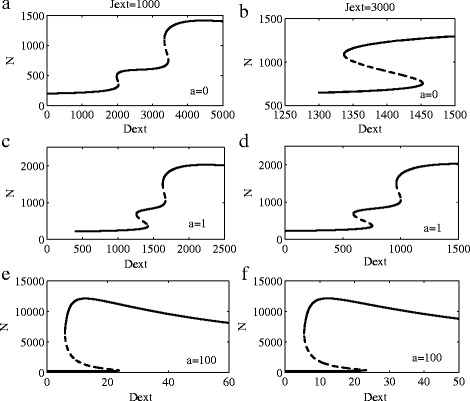
Bifurcation diagrams for the levels of different proteins. **a**-**b**: Bifurcation diagrams for the one-cell Notch1-Dll1-Jag1 circuit. **c**-**f**: Bifurcation diagrams for the one-cell Notch1-Dll1-Jag1-Fringe circuit. **a**-**c**-**e**: Bifurcation diagrams of Notch1 protein levels when driven by external Dll1 at *J*_*ext*_=1000. **b**-**d**-**f**: Bifurcation diagrams of Notch1 protein levels when driven by external Dll1 for fixed level of *J*_*ext*_=3000

**Fig. 4:**
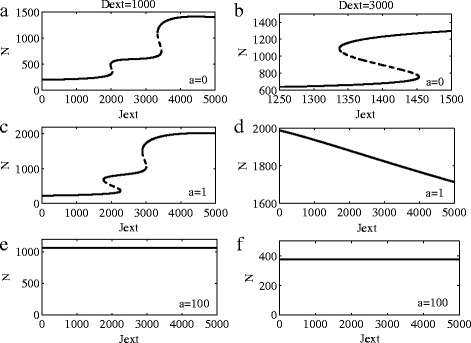
Bifurcation diagrams. **a**-**b**: Bifurcation diagrams for the one-cell Notch1-Dll1-Jag1 circuit. **c**-**f**: Bifurcation diagrams for the one-cell Notch1-Dll1-Jag1-Fringe circuit. **a**-**c**-**e**: Bifurcation diagrams of Notch1 protein levels when driven by external Jag1 at *D*_*ext*_=1000. **b**-**d**-**f**: Bifurcation diagrams of Notch1 protein levels when driven by external Jag1 at *D*_*ext*_=3000

Values for Figs. 3 and 4

10 $$\begin{array}{*{20}l} {}\frac{{dD}_{i}}{dt}&=\frac{I_{0}^{2}}{I_{i}^{2}+I_{0}^{2}}D_{0}-\left(1+\frac{a [L]^{n}}{k_{1}^{n}+[L]^{n}}\right)\\ &\quad\times(k_{c}D_{i}N_{i}+k_{t}D_{i}\left\langle N_{j}\right\rangle_{i})-\gamma D_{i}, \end{array} $$

11 $$\begin{array}{*{20}l} {}\frac{{dJ}_{i}}{dt}&=\left(1+\frac{I_{i}^{5}}{I_{i}^{5}+I_{0}^{5}}\right)J_{0}-\frac{k_{2}^{n}}{k_{2}^{n}+[L]^{n}}\\ &\quad\times(k_{c}J_{i}N_{i}+k_{t}J_{i}\left\langle N_{j}\right\rangle_{i})-\gamma J_{i}, \end{array} $$

12 $$\begin{array}{*{20}l} {}\frac{{dI}_{i}}{dt}&=\left(1+\frac{a [L]^{n}}{k_{1}^{n}+[L]^{n}}\right)k_{t}N_{i}\left\langle D_{j}\right\rangle_{i}\\ &\quad+\frac{k_{2}^{n}}{k_{2}^{n}+[L]^{n}}k_{t}N_{i} \left\langle J_{j}\right\rangle_{i}-\gamma_{I}I. \end{array} $$

The notations 〈*D*_*j*_〉_*i*_, 〈*J*_*j*_〉_*i*_ and 〈*N*_*j*_〉_*i*_ refer to the average levels of all *j* neighbors of the *i*-th Dll1, Jag1, and Notch1, respectively.

Several studies have reported abnormal activity of Notch1 in human brain tumors. But it is still not clear how Notch1 signaling pathway affects the occurrence and maintenance of gliomas. In the following sections, based on bifurcation analysis of the models, we will analyze how Notch1 signaling pathway modulates glioma cell fate decisions.

## Results and discussion

### Effect of Dll1-Jag1-Fringe on cell fate decisions for one-cell system

Gliomas may produce neural stem cells which can then differentiate into neurons or glial cells at all stages of tumorigenesis at maturity. Therefore, it could be argued that gliomas produced by cells with different maturity level can show different expression of Notch1 signal cascade of spectrum, which reflects the origin of gliomas [26]. These expression products can also be used to identify different grades of gliomas, including primary and secondary gliomas. Studies have shown correlation of Notch1 expression and glioma grades [27–29].

Immunohistochemistry of a primary human glioma tissue array shows the presence of the Notch1 intracellular domain in the nucleus, indicating Notch1 activation in situ. Down-regulation of Notch1, Dll1, or Jag1 by RNA interference can induce apoptosis or inhibit proliferation in multiple glioma cell lines. Notch1 and its ligands may present novel therapeutic targets in the treatment of gliomas [18]. Preliminary works in laboratory from phage display biopanning on human glioma cells resulted in the isolation of two peptides that share significant homology to regions of Jag1 and Dll1, two Notch1 receptor ligands. These findings suggested the presence of Notch1 on human glioma cells, which was further supported by cDNA microarray data. All these findings prompt us to study the biological relevance of Notch signaling to the glioma cell fate decisions.

We explore the effects of Dll1-Jag1-Fringe on glioma cell fate decisions by analyzing the model (5)-(8). The effect of ligand Jag1 and Fringe is shown in Fig. 3. As we can see from (a) and (b), for the case of no Fringe, when the value of Jag1 becomes more larger, the system changes from twice transitions to only once. The difference between (a) and (c) is the value of *a* (on behalf of Fringe existence), which represents the presence of Fringe, but only a small intensity. Stable steady states almost do not change. When the *a* value is further increased, the transition becomes only once.

The effects of ligand Dll1 and Fringe on the system dynamics are shown in Fig. 4. The standard values of all parameters are listed in Table 1. As we can see from (a) and (b), for the case of no Fringe, when the value of Dll1 becomes more larger, the system changes from twice state transitions to only once. However, when the Fringe regulation is large enough, we can see that on state transitions occur even when *J*_*ext*_ is small enough. Compared with Fig. 3, The influence of Dll1 on the system is more larger than Jag1.

From Figs. 3 and 4, preliminary conclusions can be obtained as follows: (1) in the Notch1 signaling system of gliomas, the impact of Dll1 is greater than Jag1; (2) expression of the Dll1 ligand is shown to be increased in gliomas when compared with normal brain tissue; and (3) the appearance of Fringe will change the state transtions in the Notch1 signaling system of gliomas.

### Effect of Dll1-Jag1-Fringe on cell fate decisions for the two-cell system

**Fig. 5:**
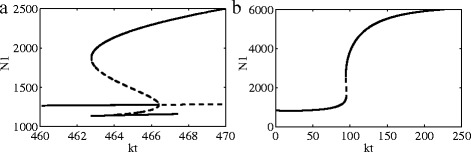
Dynamical properties of Notch1-Dll1 signaling circuit. **a** Two cells are fully symmetrical. **b** The symmetry of two cells is broken. In Eq. (14), the term *k*_*c*_ *D*_2_ is replaced by 3×*k*_*c*_ *D*_2_

To make a breakthrough and research into the impact of the Notch1 signaling pathway more deeply, we describe the two-cell model of the three cases: Notch1-Dll1 only (N-D), Notch1-Dll1-Jag1 (N-D-J), and the model including the Fringe (N-D-J-F). It has been experimentally shown that given the expression of Dll1 in primary human gliomas, efficient Dll1 were transfected into six glioma lines and their effects assessed. Dll1 knockdown produced dramatic effects, inducing a spindleshaped morphology initially (not shown) with subsequent cell death. Significant decreases in viable cell number were evident in all six glioma cell lines as evaluated by alamarBlue assay. We first evaluate the dynamics of N-D signaling for the two-cell system (13)-(18). Bifurcation diagrams with *k*_*t*_ as a control parameter is showed in Fig. 5. The standard values of all parameters are shown in Table 2.

13 $$\begin{array}{*{20}l} \frac{{dN}_{1}}{dt}&=\left(1+\frac{I_{1}^{2}}{I_{1}^{2}+I_{0}^{2}}\right)N_{0}-N_{1}(k_{c}D_{1}+k_{t}D_{2})-\gamma N_{1}, \end{array} $$

**Table 2:** Standard parameter values in the model (13)-(18)

Parameters	Definitions	Values	Unit
*N*_0_	The innate production rates of Notch1	500	Number of proteins
*D*_0_	The innate production rates of Dll1	500	Number of proteins
*I*_0_	The innate production rates of NICD	200	Number of proteins
*γ*	The degradation rate of proteins	0.1	time ^−1^(*h* ^−1^)
	Notch1, Jag1, and Dll1		
*γ*_*I*_	The degradation rate of NICD	0.5	time ^−1^(*h* ^−1^)
*k*_*t*_	The strengths of trans-activation	$ (4.7\times 10^{-5})^{\divideontimes },$	time ^−1^(*h* ^−1^)
		(10^−5^)^∗^	
*k*_*c*_	The strengths of cis-activation	6.1×10^−4^	time ^−1^(*h* ^−1^)

$\divideontimes $ Values for Fig. 5 a. *Values for Fig. 5 b

14 $$\begin{array}{*{20}l} \frac{{dN}_{2}}{dt}&=\left(1+\frac{I_{2}^{2}}{I_{2}^{2}+I_{0}^{2}}\right)N_{0}-N_{2}(k_{c}D_{2}+k_{t}D_{1})-\gamma N_{2}, \end{array} $$

15 $$\begin{array}{*{20}l} \frac{{dD}_{1}}{dt}&=\frac{I_{0}^{2}}{I_{1}^{2}+I_{0}^{2}}D_{0}-D_{1}(k_{c}N_{1}+k_{t}N_{2})-\gamma D_{1}, \end{array} $$

16 $$\begin{array}{*{20}l} \frac{{dD}_{2}}{dt}&=\frac{I_{0}^{2}}{I_{2}^{2}+I_{0}^{2}}D_{0}-D_{2}(k_{c}N_{2}+k_{t}N_{1})-\gamma D_{2}, \end{array} $$

17 $$\begin{array}{*{20}l} \frac{{dI}_{1}}{dt}&=k_{t}N_{1}D_{2}-\gamma_{I}I_{1}, \end{array} $$

18 $$\begin{array}{*{20}l} \frac{{dI}_{2}}{dt}&=k_{t}N_{2}D_{1}-\gamma_{I}I_{2}. \end{array} $$

**Fig. 6:**
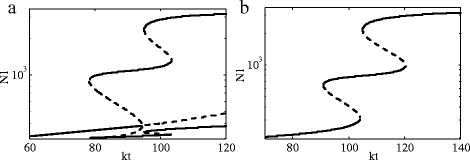
Bifurcation diagram of Notch1-Dll1-Jag1 signaling circuit. **a** Two cells are fully symmetrical. **b** The symmetry of two cells is broken. The term *k*_*c*_ *J*_2_ is replaced by 1.2×*k*_*c*_ *J*_2_ in Eq. (20) and and the term *k*_*t*_ *N*_1_ is replaced by 4×*k*_*t*_ *N*_1_ in Eq. (22)

Similarity, it has been shown that Jag1 knockdown can slow growth significantly in several of the glioma lines. Effects of Jag1 on glioma cells can also be assessed. We then analyze the dynamics of N-D-J signaling for two-cell system (19)-(26). The bifurcation diagrams are shown in Fig. 6. The standard parameter values are listed in Table 3.

19 $$\begin{array}{*{20}l} \frac{{dN}_{1}}{dt}&=\left(1+\frac{I_{1}^{2}}{I_{1}^{2}+I_{0}^{2}}\right)N_{0}-N_{1}(k_{c}D_{1}+k_{c}J_{1})\\ &\quad-N_{1}(k_{t}D_{2}+k_{t}J_{2}) -\gamma N_{1}, \end{array} $$

**Table 3:** Standard parameter values in the model (19)−(26)

Parameters	Definitions	Values	Unit
*N*_0_	The innate production rates of Notch1	1600	Number of proteins
*D*_0_	The innate production rates of Dll1	1800	Number of proteins
*J*_0_	The innate production rates of Jag1	1200	Number of proteins
*I*_0_	The innate production rates of NICD	200	Number of proteins
*γ*	The degradation rate of proteins	0.1	time ^−1^(*h* ^−1^)
	Notch1, Jag1, and Dll1		
*γ*_*I*_	The degradation rate of NICD	$0.6^{\divideontimes },0.5^{*}$	time ^−1^(*h* ^−1^)
*k*_*t*_	The strengths of trans-activation	$ (7\times 10^{-6})^{\divideontimes },$	time ^−1^(*h* ^−1^)
		(10^−5^)^∗^	
*k*_*c*_	The strengths of cis-activation	$ (4\times 10^{-4})^{\divideontimes },$	time ^−1^(*h* ^−1^)
		(6.1×10^−4^)^∗^	

$\divideontimes $Values for Fig. 6 a. *Values for Fig. 6 b

20 $$\begin{array}{*{20}l} \frac{{dN}_{2}}{dt}&=\left(1+\frac{I_{2}^{2}}{I_{2}^{2}+I_{0}^{2}}\right)N_{0}-N_{2}(k_{c}D_{2}+k_{c}J_{2})\\ &\quad-N_{2}(k_{t}D_{1}+k_{t}J_{1}) -\gamma N_{2}, \end{array} $$

21 $$\begin{array}{*{20}l} \frac{{dD}_{1}}{dt}&=\frac{I_{0}^{2}}{I_{1}^{2}+I_{0}^{2}}D_{0}-D_{1}(k_{c}N_{1}+k_{t}N_{2})-\gamma D_{1}, \end{array} $$

22 $$\begin{array}{*{20}l} \frac{{dD}_{2}}{dt}&=\frac{I_{0}^{2}}{I_{2}^{2}+I_{0}^{2}}D_{0}-D_{2}(k_{c}N_{2}+k_{t}N_{1})-\gamma D_{2}, \end{array} $$

23 $$\begin{array}{*{20}l} \frac{{dJ}_{1}}{dt}&=\left(1+\frac{I_{1}^{5}}{I_{1}^{5}+I_{0}^{5}}\right)J_{0}-J_{1}(k_{c}N_{1}+k_{t}N_{2})-\gamma J_{1}, \end{array} $$

24 $$\begin{array}{*{20}l} \frac{{dJ}_{2}}{dt}&=\left(1+\frac{I_{2}^{5}}{I_{2}^{5}+I_{0}^{5}}\right)J_{0}-J_{2}(k_{c}N_{2}+k_{t}N_{1})-\gamma J_{2}, \end{array} $$

25 $$\begin{array}{*{20}l} \frac{{dI}_{1}}{dt}&=k_{t}N_{1}D_{2}+k_{t}N_{1}J_{2}-\gamma_{I}I_{1}, \end{array} $$

26 $$\begin{array}{*{20}l} \frac{{dI}_{2}}{dt}&=k_{t}N_{2}D_{1}+k_{t}N_{2}J_{1}-\gamma_{I}I_{2}. \end{array} $$

Current data has shown, along with Notch1 expression, the expression of the Notch1 ligands, Dll1 and Jag1, in both glioma cell lines. To our knowledge, this is only the second example described in the literature of Notch1 ligand overexpression in the human malignant disease, with a previous report in cervical cancer. Figures 5 and 6 show a critial role of Dll1 and Jag1 in glioma cells, which reflect the relatively greater efficiency of Dll1 than Jag1 but also indicate a greater role for Dll1 than Jag1 as a Notch1 ligand in glioma cells. At the same time, we can know that the expression of Dll1 increases with increased Notch1 expression. In contrast, the expression of Jag1 has an inverse relationship with the expression of Notch1.

**Fig. 7:**
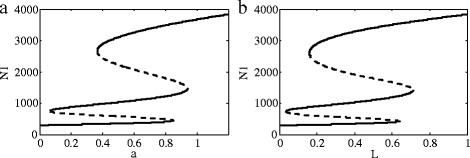
Bifurcation diagrams for the effect of Fringe protein. **a** The bifurcation with *a* as a control parameter at *L*=1. The value *a* reflects the effect of Fringe on cis-inhibition and trans-activation between Notch1 and Dll1. The term *k*_*c*_ *N*_2_ *J*_2_ is replaced by 1.2×*k*_*c*_ *N*_2_ *J*_2_ in Eq. (28) and the term *k*_*t*_ *D*_1_ *N*_2_ is replaced by 42×*k*_*t*_ *D*_1_ *N*_2_ in Eq. (29). As *a* gradually increases, the healthy tissue changes from normal to grade I ∼ II of gliomas. When *a* is increased to a certain value, the state will switch from grade I ∼ II to grade III ∼ IV of gliomas. **b** The bifurcation diagram with *L* as a control parameter at *a*=1.2, which reflects the dynamics of Notch1 signaling pathway after the addition of Lunatic Fringe. When the Fringe is inhibited, the transition from gliomas to a healthy state can be gradually realized

Finally, we analyze the dynamics of Notch1-Dll1-Jag1-Fringe signaling for two-cell system (27)-(34). The dynamics of Notch1 signaling pathway after the addition of Fringe is shown in Fig. 7. The standard parameter values are shown in Table 4. To measure the effect of the Fringe on ligand-induced Notch1 signaling, scholars measured its ability to modulate signaling induced by either Dll1 or Jag1 in 3T3 cells ectopically expressing Notch1 by using a CSL-reporter coculture assay [30]. Consistent with previous findings, LFng potentiated CSL-reporter activity induced by Dll1 and suppressed CSL-reporter activity by Jag1. These findings suggest that Fringe can modulate Notch1 signaling by regulating both Dll1 and Jag1. The link between glycosylation and Fringe activity was also assessed by using mutant cells defective in transferring fucose to proteins. Co-culture of Notch1-expressing and Jag1-expressing cells increased reporter gene activity. While the reporter gene expression was reduced if the Notch1-expressing cells were co-transfected with Manic or Lunatic Fringe [31].

27 $$\begin{array}{*{20}l} \frac{{dN}_{1}}{dt}&=\left(1+\frac{I_{1}^{2}}{I_{1}^{2}+I_{0}^{2}}\right)N_{0}-\left(1+\frac{a[L]^{n}}{k_{1}^{n}+[L]^{n}}\right)(k_{c}N_{1}D_{1}\\ &\quad+k_{t}N_{1}D_{2}) -\frac{k_{2}^{n}}{k_{2}^{n}+[L]^{n}}(k_{c}N_{1}J_{1}+k_{t}N_{1}J_{2})-\gamma N_{1}, \end{array} $$

**Table 4:** Standard parameter values in the model (27)−(34)

Parameters	Definitions	Values	Unit
*N*_0_	The innate production rates of Notch1	1600	Number of proteins
*D*_0_	The innate production rates of Dll1	1800	Number of proteins
*J*_0_	The innate production rates of Jag1	1200	Number of proteins
*I*_0_	The innate production rates of NICD	200	Number of proteins
*γ*	The degradation rate of proteins	0.1	time ^−1^(*h* ^−1^)
	Notch1, Jag1, and Dll1		
*γ*_*I*_	The degradation rate of NICD	0.5	time ^−1^(*h* ^−1^)
*k*_*t*_	The strengths of trans-activation	7×10^−6^	time ^−1^(*h* ^−1^)
*k*_*c*_	The strengths of cis-activation	6.1×10^−4^	time ^−1^(*h* ^−1^)

Values for Fig. 7

28 $$\begin{array}{*{20}l} \frac{{dN}_{2}}{dt}&=\left(1+\frac{I_{2}^{2}}{I_{2}^{2}+I_{0}^{2}}\right)N_{0}-\left(1+\frac{a[L]^{n}}{k_{1}^{n}+[L]^{n}}\right)(k_{c}N_{2}D_{2}\\ &\quad+k_{t}N_{2}D_{1}) -\frac{k_{2}^{n}}{k_{2}^{n}+[L]^{n}}(k_{c}N_{2}J_{2}+k_{t}N_{2}J_{1})-\gamma N_{2}, \end{array} $$

29 $$\begin{array}{*{20}l} \frac{{dD}_{1}}{dt}&=\frac{I_{0}^{2}}{I_{1}^{2}+I_{0}^{2}}D_{0}-\left(1+\frac{a [L]^{n}}{k_{1}^{n}+[L]^{n}}\right)\\ &\quad\times(k_{c}D_{1}N_{1}+k_{t}D_{1}N_{2})-\gamma D_{1}, \end{array} $$

30 $$\begin{array}{*{20}l} \frac{{dD}_{2}}{dt}&=\frac{I_{0}^{2}}{I_{2}^{2}+I_{0}^{2}}D_{0}-\left(1+\frac{a [L]^{n}}{k_{1}^{n}+[L]^{n}}\right)\\ &\quad\times(k_{c}D_{2}N_{2}+k_{t}D_{2}N_{1})-\gamma D_{2}, \end{array} $$

31 $$\begin{array}{*{20}l} \frac{{dJ}_{1}}{dt}&=\left(1+\frac{I_{1}^{5}}{I_{1}^{5}+I_{0}^{5}}\right)J_{0}-\frac{k_{2}^{n}}{k_{2}^{n}+[L]^{n}}\\ &\quad\times(k_{c}J_{1}N_{1}+k_{t}J_{1}N_{2})-\gamma J_{1}, \end{array} $$

32 $$\begin{array}{*{20}l} \frac{{dJ}_{2}}{dt}&=\left(1+\frac{I_{2}^{5}}{I_{2}^{5}+I_{0}^{5}}\right)J_{0}-\frac{k_{2}^{n}}{k_{2}^{n}+[L]^{n}}\\ &\quad\times(k_{c}J_{2}N_{2}+k_{t}J_{2}N_{1})-\gamma J_{2}, \end{array} $$

33 $$\begin{array}{*{20}l} \frac{{dI}_{1}}{dt}&=\left(\!1+\frac{a [L]^{n}}{k_{1}^{n}+[L]^{n}}\right)k_{t}N_{1}D_{2}+\frac{k_{2}^{n}}{k_{2}^{n}+[L]^{n}}k_{t}N_{1}J_{2}-\gamma_{I}I_{1}, \end{array} $$

34 $$\begin{array}{*{20}l} {}\frac{{dI}_{2}}{dt}&=\left(\!1+\frac{a [L]^{n}}{k_{1}^{n}+[L]^{n}}\right)k_{t}N_{2}D_{1}+\frac{k_{2}^{n}}{k_{2}^{n}+[L]^{n}}k_{t}N_{2}J_{1}-\gamma_{I}I_{2}. \end{array} $$

## Conclusions

Glioma is one of the worst tumors of of central nervous system. The treatment difficulty lies in the relapse, which is related with glioma cells proliferation and invasive growth. In recent years, the Notch1 signaling in proliferation of gliomas for the survival is increasingly concerned. It has been shown the over expression of Notch1 protein in my kinds of cancers, such as skin, lung, and and other caners. Notch1 signaling pathway plays important roles in cell proliferation, differentiations, and apoptosis. Tumor gene therapy and the development of new drugs using Notch1 receptors as targets will open new areas for the tumor therapy. Preliminary research has shown that Notch1 has a good application prospect as an anti-tumor target. Our results confirmed that the regulation between Notch1, its ligands, and Fringe can modulate the glioma cell fate decisions. For the two-cell system, a pitchfork bifurcation occurs due to the symmetry of two cells. Once breaking the symmetry, saddle node bifurcations occur, which is similar to the situation occurred in the single cell system. More importantly, we show that Fringe can modulate the glioma cell fate decisions by regulating the Notch1 signaling, i.e., realizing the transition of grades III ∼ IV to grades I ∼ II, and then to normal brain tissue. Besides Fringe, Dll1 and Jag1 also play critical roles in glioma cell fate decisions due to combinatorial effects between them.

Although our model provides a new theoretical framework to investigate the effects of Dll1, Jag1 and Fringe in the Notch1 signaling system in glioma cells, it ignores the spatial effects which can be also important. Other limitations of our model include: no distinction between soluble and membrane-bound ligands, no time delays between existence of fringe and its action on Notch1 signaling pathway, and no difference between the Fringe family members. However, the model still presents the first step toward the possible reasons of over expression of Notch1 in gliomas cells, e.g., high Fringe expression, which provides us some possible clinic treatment of gliomas, e.g., inhibition of Fringe expression.

## References

[1] Bray SJ (2006). Notch signalling: A simple pathway becomes complex. Nat Rev Mol Cell Biol.

[2] Andersson SJ, Sandberg R, Lendahl U (2011). Notch signaling: Simplicity in design, versatility in function. Development.

[3] Bolós V, Grego-Bessa J, de la Pompa JL (2007). Notch signaling in development and cancer. Endocr Rev.

[4] Srividhya J. A mathematical model for inter-cellular inductive. 2012; 2(3):102–7.

[5] Wang R, Liu K, Chen L, Aihara K (2011). Neural fate decisions mediated by trans-activation and cis-inhibition in notch signaling. Bioinformatics.

[6] LeBon L, Lee TV, Sprinzak D, Jafar-Nejad H, Elowitz MB (2014). Fringe proteins modulate notch-ligand cis and trans interactions to specify signaling states. eLife.

[7] Kato TM, Kawaguchi A, Kosodo Y, Niwa H, Matsuzaki F (2010). Lunatic fringe potentiates notch signaling in the developing brain. Mol Cell Neurosci.

[8] Blair SS (2000). Notch signaling: Fringe really is a glycosyltransferase. Curr Biol.

[9] Yang LT, Nichols JT, Yao C, Manilay JO, Robey EA, Weinmaster G (2005). Fringe glycosyltransferases differentially modulate notch1 proteolysis induced by delta1 and jagged1. Mol Biol Cell.

[10] Chen J, Lu L, Shi S, Stanley P (2006). Expression of notch signaling pathway genes in mouse embryos lacking *β*4*g**a**l**a**c**t**o**s**y**l**t**r**a**n**s**f**e**r**a**s**e*−1. Gene Expr Patterns.

[11] Johnston SH, Rauskolb C, Wilson R, Prabhakaran B, Irvine KD, Vogt TF (1997). A family of mammalian fringe genes implicated in boundary determination and the notch pathway. Development.

[12] Artavanis-Tsakonas S, Rand MD, Lake RJ (1999). Notch signaling: cell fate control and signal integration in development. Science.

[13] Miele L, Osborne B (1999). Arbiter of differentiation and death: Notch signaling meets apoptosis. J Cell Physiol.

[14] Oda T, Elkahloun AG, Pike BL, Okajima K, Krantz ID, Genin A, Piccoli DA, Meltzer PS, Spinner NB, Collins FS, Chandrasekharappa SC (1997). Mutations in the human jagged1 gene are responsible for alagille syndrome. Nat Genet.

[15] Li L, Krantz ID, Deng Y, Genin A, Banta AB, Collins CC, Qi M, Trask BJ, Kuo WL, Cochran J, Costa T, Pierpont ME, Rand EB, Piccoli DA, Hood L, Spinner NB (1997). Alagille syndrome is caused by mutations in human jagged1, which encodes a ligand for notch1. Nat Genet.

[16] Gray GE, Mann RS, Mitsiadis E, Henrique D, Carcangiu ML, Banks A, Leiman J, Ward D, Ish-Horowitz D, Artavanis-Tsakonas S (1999). Human ligands of the notch receptor. Am J Pathol.

[17] LaVoie MJ, Selkoe DJ (2003). The notch ligands, jagged and delta, are sequentially processed by *α*-secretase and presenilin *γ*-secretase and release signaling fragments. J Biol Chem.

[18] Purow BW, Haque RM, Noel MW, Su Q, Burdick MJ, Lee J, Sundaresan T, Pastorino S, Park JK, Mikolaenko I, Maric D, Eberhart CG, Fine HA (2005). Expression of notch-1 and its ligands, delta-like-1 and jagged-1, is critical for glioma cell survival and proliferation. Cancer Res.

[19] YU C, Yin C, LV S (2008). Notch-1 signaling pathway regulates proliferation and differentiation of human brain glioma stem cells. Chinese J Minim Invasive Neurosurg.

[20] Hai-Bo Y I Y. W. Z. e. a. ShiSS (2006). The expression and significance of notch-1 gene in human gliomas. Cancer Res Prevention & Treatment.

[21] Collier JR, NAM Monk, PK Maini, JH Lewis (1996). Pattern formation by lateral inhibition with feedback: a mathematical model of delta-notch intercellular signaling. J Theor Biol.

[22] Özbudak EM, Lewis J (2008). Notch signalling synchronizes the zebrafish segmentation clock but is not needed to create somite boundaries. PLoS Genet..

[23] Sprinzak D, Lakhanpal A, Lebon L, Santat LA, Fontes ME, Anderson GA, Garcia-Ojalvo J, Elowitz MB (2010). Cis-interactions between notch and delta generate mutually exclusive signalling states. Nature.

[24] Boareto M, Jolly MK, Lu M, Onuchic JN, Clementi C, Ben-Jacob E (2015). Jagged-delta asymmetry in notch signaling can give rise to a sender/receiver hybrid phenotype proc. Natl Acad Sci.

[25] Sprinzak D, Lakhanpal A, Lebon L, Santat LA, Fontes ME, Anderson GA, Garcia-Ojalvo J, Elowitz MB (2010). Cis-interactions between notch and delta generate mutually exclusive signalling states. Nature.

[26] Stockhausen MT, Kristoffersen K, Poulsen HS (2012). The functional role of Notch signaling in human gliomas. Neuro-Oncol.

[27] Phillips HS, Kharbanda S, Chen R, Forrest WF, Soriano RH, Wu TD, Misra A, Nigro JM, Colman H, Soroceanu L, Williams PM, Modrusan Z, Feuerstein BG, Aldape K (2006). Molecular subclasses of highgrade glioma predict prognosis, delineate a pattern of disease progression, and resemble stages in neurogenesis. Cancer Cell.

[28] Ladi E, Nichols JT, Ge W, Miyamoto A, Yao C, Yang LT, Boulter J, Sun YE, Kintner C, Weinmaster G (2005). The divergent dsl ligand dll3 does not activate notch signaling but cell autonomously attenuates signaling induced by other dsl ligands. J Cell Biol.

[29] Tso CL, Shintaku P, Chen J, Liu Q, Liu J, Chen Z, Yoshimoto K, Mischel PS, Cloughesy TF, Liau LM, Nelson SF (2006). Primary glioblastomas express mesenchymal stem-like properties. Mol Cancer Res.

[30] Hicks C, Johnston SH, diSibio G, Collazo A, Vogt TF, Weinmaster G (2000). Fringe differentially modulates jagged1 and delta1 signaling through notch1 and notch2. Nat Cell Biol.

[31] Moloney DJ, VM Panin, SH Johnston, J Chen (2000). Fringe is a glycosyltransferase that modifies notch. Nature.

